# Improving physical health form completion on a general inpatient adult ward

**DOI:** 10.1192/bjo.2021.607

**Published:** 2021-06-18

**Authors:** Brandon Wong, Anjna Vekaria

**Affiliations:** CNWL

## Abstract

**Aims:**

The government's Five Year Forward View Plan for Mental Health has set a target for 280,000 people with severe mental health problems to be offered screening and appropriate intervention based on physical health risk stratification, including obesity, diabetes and heart disease. As such, physical health review for patients on a general inpatient adult psychiatry ward includes routine blood tests for cholesterol levels and HbA1c. They are recorded together in a Physical Health (PH) Form in the patient's electronic record and used to stratify cardiovascular risk factors and risk of diabetes. If a patient declines these blood tests it should be recorded on the PH form.

This study aims to improve the completion of Physical Health forms to ≥95% by within a 4-month period on a general adult inpatient psychiatric ward.

**Method:**

PH form completion was measured using Tableau Software for a 4-week period as a baseline then fortnightly during the study. PH form completion required HbA1c and cholesterol levels to be inputted, or to be marked as declined where the patient had declined these tests. Potential interventions were discussed by clinicians and implemented using PDSA cycles with iterative changes tested and analysed. PH form completion was re-audited monthly for a 6-month period.

**Result:**

Baseline data showed 61.54% of patients had physical health forms completed (n = 26; 61.54% with HbA1c, 76.92% with cholesterol completed). Iterative changes and improvements included; (i) paper list to track PH form completion, (ii) table on Microsoft Word, (iii) Excel spreadsheet, and (iv) a conditionally formatted Excel spreadsheet. The conditionally formatted Excel spreadsheet was colour-coded to show completed elements as green and incomplete elements as red.

Paper lists increased PH completion to 84.85% (n = 33). Word table increased PH completion to 96.43% (n = 28). Excel spreadsheet had PH completion of 96.67% (n = 30). Colour coded excel spreadsheet increased PH completion to 100% (n = 28). This was used as standard practice with sustained 100% completion in November (n = 34) and December (n = 39). The improvement was sustained to January 2021, although there was a decrease to 97.7% (n = 30).

**Conclusion:**

It was hypothesised an intervention to track completion of PH forms would improve completion rate. The use of a colour-coded conditionally formatted Excel spreadsheet improved PH form completion to 100% within an 8-week period and a sustained increase of >95% 6 months after the study began. This study recommends the use of such an electronic record keeping system to assist with PH form completion.

